# The Gut–Organ-Axis Concept: Advances the Application of Gut-on-Chip Technology

**DOI:** 10.3390/ijms24044089

**Published:** 2023-02-17

**Authors:** Yuxi Guo, Xuefeng Chen, Pin Gong, Guoliang Li, Wenbo Yao, Wenjuan Yang

**Affiliations:** School of Food and Biological Engineering, Shaanxi University of Science and Technology, Xi’an 710021, China

**Keywords:** organ-on-chip, gut–organ-axis, gut–organ-on-chip, bio-inspired microfluidic, disease

## Abstract

The intestine is considered to be a vital digestive organ to absorb nutrients and is the largest immune organ, while numerous microorganisms coexist with the host. It is well known that the complex interactions between the gut microbiota and the host’s immune system inevitably affect the function of other organs, creating an “axis” between them. During the past few years, a new technique based mainly on microfluidics and cell biology has been developed to emulate the structure, function, and microenvironment of the human gut, called the “gut-on-chip”. This microfluidic chip provides insight into key aspects of gut function in health and disease, such as the gut–brain axis, gut–liver axis, gut–kidney axis, and gut–lung axis. In this review, we first describe the basic theory of the gut axis and the various composition and parameter monitoring of the gut microarray systems, as well as summarize the development and emerging advances in the gut–organ-on-chip, with a focus on the host-gut flora and nutrient metabolism, and highlight their role in pathophysiological studies. In addition, this paper discusses the challenges and prospects for the current development and further use of the gut–organ-on-chip platform.

## 1. Introduction

The intestine is utilized to be responsible for facilitating the digestion and absorption of nutrients. As the largest immune organ in the body, it plays an invaluable role in sustaining normal immune defense [1]. The human gut supports a positive micro-ecological environment for micro-organisms and has metabolic functions that the human body does not possess on its own [1]. The typical adult gut contains over 1 kg of bacteria and is a highly diverse and dynamic ecosystem that weighs essentially the same as the human brain. There are approximately 10^13^–10^14^ microorganisms in the gut, containing more than 1000 times the number of genes in the genome and is more complex than brain [2].

The gut is currently assumed to play a central role in sustaining the normal function of other organs and in the pathogenesis of many diseases [3,4,5,6,7,8,9]. Evidence from recent studies describes bidirectional interactions between the gut microbiota and other organs, providing theoretical support for an integrated model that integrates multiple organs, the gut, and immune system with the gut microbiota [10,11,12]. The last five years have seen a paradigm shift in our comprehension of the gut–brain axis [13], the gut–liver axis [10], the gut–kidney axis [12], and the gut–lung axis [14]. Intestinal disruption or an imbalance of the intestinal flora as well as an impaired structure and function of the gut, along with an imbalance in immune homeostasis [15], lead to a range of intestinal diseases such as IBD [16], Crohn’s disease, and other organ diseases [17]. Over the past 30 years, the study of known host–microbe interactions has received extensive attention and has developed rapidly. However, many of the regulatory mechanisms underlying this complex process remain unclear. To address this complexity, a simplified approach is needed that reduces both the host and microbiome to a level where experimental variables can be tightly controlled and intentionally manipulated [18].

**Figure 1:**
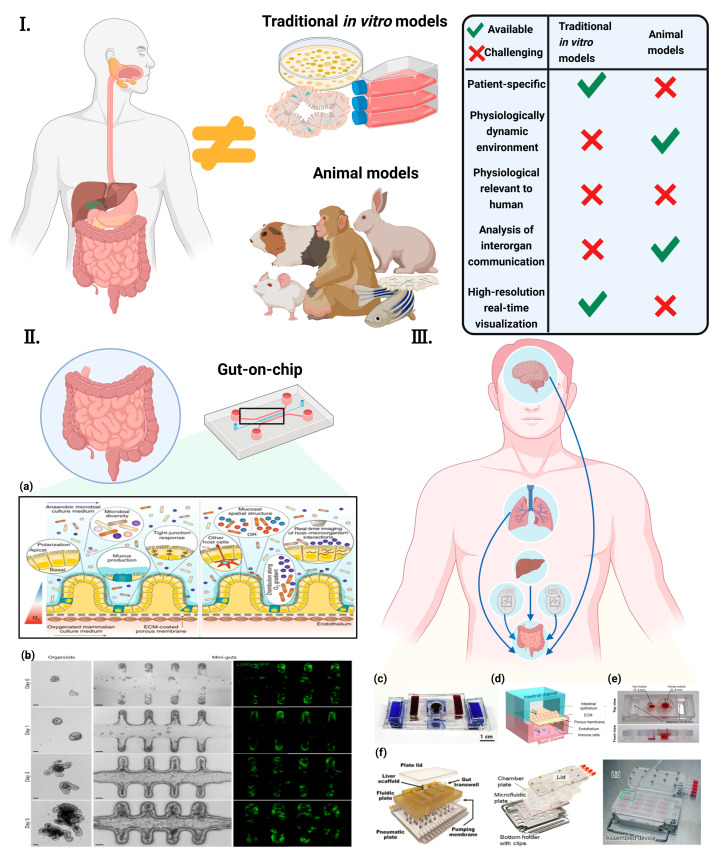
Concept of “gut-axis” organ-on-a-chip (**I**) Conventional in vitro models and animal models are physiologically different from the human body. They also hinder the understanding of human diseases and the development of new therapeutic strategies [27] ©Copyright 2021, MDPI. (**II**) The concept of the GoC simulates the dynamic 3D microenvironment of an organ on a small scale. (**a**) Human-gut-microbiome on a chip [28] ©Copyright 2019, Nature.; (**b**) Establishment of long-term homeostatic culture of tubular mini-guts [29] ©Copyright 2020, Nature. (**III**) “Gut-axis” organ-on-a-chip. (**c**) Gut–brain axis on chip [30] ©Copyright 2021, Elsevier. (**d**) Gut–lung axis on chip [31] (**e**) Gut–kidney axis on chip [32] ©Copyright 2021, Elsevier. (**f**) Gut–liver axis on chip [33] ©Copyright 2017, Wiley [34] ©Copyright 2021, Nature. [35] ©Copyright 2020, Royal Soc Chemistry.

In general, animal models are the primary method for studying the gut and its associated diseases in vivo [19]. However, these models are limited by their inability to faithfully reflect human physiology [20,21]. In many cases, particularly in drug response studies, animal models are unable to fully replicate or simulate the complex responses observed in humans [22]. In addition, the toxic properties of drugs often vary considerably between species, and compounds that are toxic to humans may not be toxic to animals, and vice versa [21]. In addition to animal models, in vitro models are also extensively used to study the gut [22]. As with animal models, these in vitro systems lack the significant characterization of physiological processes. Most in vitro models are dependent on a two-dimensional (2D) cell culture, which is inherently limiting as it does not recapitulate three-dimensional (3D) structures and local tissue interactions [18,23]. In order to properly study the intestinal physiology, pathology, or pharmacology, 3D models and dynamic culture systems must be used [24]. Although in vitro experiments and animal models have contributed enormously to our knowledge of physiology and disease and to the development of new drugs, researchers have long been aware of the frequent inconsistencies between animals, and there is an urgent need for suitable models and testbeds to better predict human responses [25]. Microfluidics-based Organ-on-a-Chip (OoC) technology, also known as a microphysiological system that mimics the physiology and function of human organs on a chip, is envisioned to integrated into the drug development pipeline. Additionally, OoC bridges the gap between animal studies and clinical trials involving human subjects [22,26] (Figure 1).

**Figure 2:**
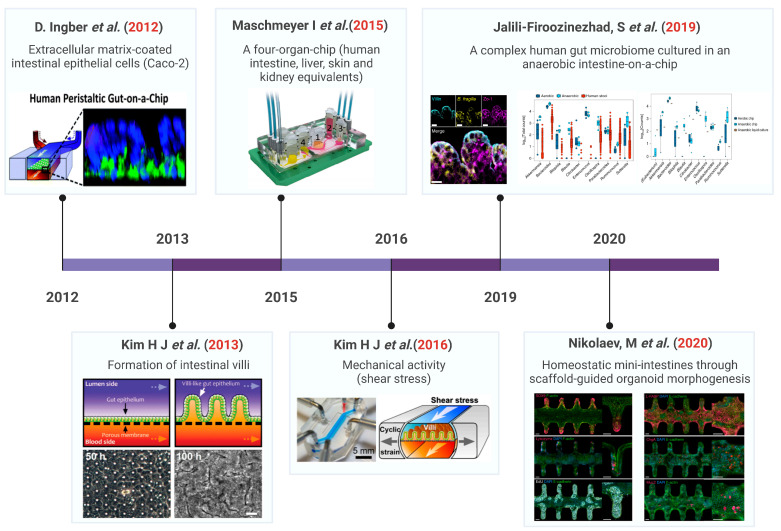
Development timeline of the gut-on-chip technology. Ref. [18] ©Copyright 2012 Nature. Ref. [42] ©Copyright 2013, Royal Soc Chemistry. Ref. [58] ©Copyright 2015, Royal Soc Chemistry. Ref. [40] ©Copyright 2016, Natl Acad Sciences. Ref. [44] ©Copyright 2019, Nature. Ref. [29] ©Copyright 2020, Nature.

Due to complex gut dynamics, host–microbiome interactions, and species differences, the gut-on-a-chip (GOC) system is a particularly necessary model for advancing knowledge of the gut physiology and disease etiology [36,37,38,39]. The history of the development of the gut chip is indicated in Figure 2. Currently, most intestinal microarrays rely on hollow channels with smooth surfaces, while recent approaches can generate crypt-villous structures by inducing flow or using scaffold surfaces [40]. The cell types used to populate the chip device vary from immortal cell lines [41,42] to primary tissues [43,44], pscs-derived intestinal organoid cells [45,46], or adult stem-cell-derived organoids [29]. The GOC can be flushed with liquid to alter the gut and basal conditions or to create gradients of growth factors or oxygen, microbial environments, etc. [47,48] or reconstruction of the spatial cell type partitioning typical of crypt structures [43]. This regulates the fine-tuning of the intestinal bacterial culture environment and allows repeated exposure and harvesting of microorganisms [44,48,49]. In addition, another advantage of the intra-chip intestine compared to static-like organs is the intracavitary flow and even peristaltic movement [42]. These GOC systems will help improve the understanding and treatment of common diseases such as inflammatory bowel disease (IBD) [50] and colorectal cancer [51]. The modular nature of the device on a chip also makes it possible to connect multiple chips to create multi-organ chips (MoCs), as well as “gut-organ-axis” chips [52,53,54,55]. The “gut-organ-axis” organ-on-a-chip system is a promising interdisciplinary technique for studying the secondary toxicity caused by the drug metabolism in organs such as the gut and liver [56,57].

This paper reviews the recent advances in GOC and the role of these systems in the study of pathophysiology is highlighted. In addition, current challenges and prospects for the development and further use of gut–organ-on-chip platforms are discussed.

## 2. Composition of the Gut–Organ-on-Chip

**Figure 3:**
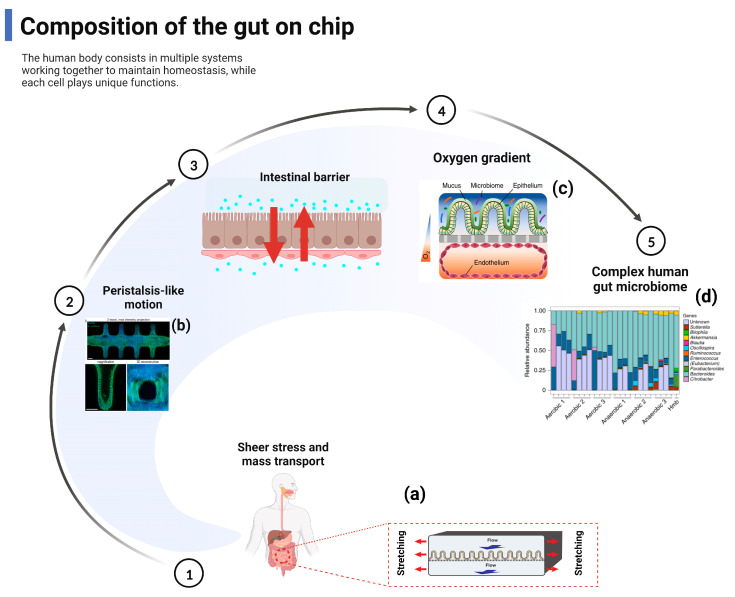
The gut-on-chip reproduces the composition and function of the gut. Main relevant reproduced include shear stress and mass transport, peristalsis-like motion, intestinal barrier, and oxygen gradient. (**a**) Gut-on-chip reveals mechanical forces impacting *Shigella* infection [49] ©Copyright 2019, Cell Press; (**b**) Bioengineering intestinal stem cell epithelia with a tubular, in vivo-like architecture [29] ©Copyright 2020, Nature; (**c**) A schematic of the two-channel gut-on-chip device with an oxygen gradient [44] ©Copyright 2019, Nature; (**d**) Differences in microbial abundance between gut-on-chip samples and a human microbiome stool sample from the Human Microbiome Project [44] ©Copyright 2019, Nature.

The organ-level physiology and function are replicated into bio-inspired microfluidic in vitro devices to overcome the limitations of animal models [18,59]. Emerging advances in the GOC system seek to replicate the inter-relationship between intestinal inflammation and host-microbiota to elucidate the pathogenesis of early intestinal disease [60,61,62]. Specifically, the GOC model aims to encapsulate key features of intestinal physiology including shear stress, mass transport, peristaltic-like motility, the intestinal barrier and oxygen gradient, and gut microbial composition, while including functional readouts to monitor biological responses (Figure 3). Under these physiological conditions, human Caco-2 intestinal epithelial cells undergo spontaneous villi morphogenesis in this mechanically dynamic intestinal microarray, unlike conventional two-dimensional cultures where cells are grown as a planar monolayer [63]. The morphogenesis of the intestinal villi on the sheet is also accompanied by the establishment of the crypt–villi axis with proliferating cells confined to the basal crypt and upward migration, drug metabolic activity, mucus secretion, and glucose reuptake [63]. Notably, due to continuous fluid flow, villi formation, and mucus production, it is also possible to co-culture living commensal microorganisms (e.g., *Lactobacillus rhamnosus* GG) [42] or a VSL#3 clinical probiotic formulation containing eight different strains of bacteria in direct contact with intestinal epithelial cells within the parenchymal channel for several weeks in vitro without affecting the barrier integrity or enterocyte function [40,42]. Indeed, the barrier function was enhanced when the intestinal epithelial cells were co-cultured with *Lactobacillus rhamnosus* gg16. Transcriptome analysis against approximately 23,000 human genes showed that in vivo-associated fluid flow and physical deformation significantly altered the gene expression profiles compared to static Transwell cultures, and mechanically active intestinal microfluidic chips, which also contained a symbiotic microbial mixture (VSL#3 probiotic formula), showing the highest genetic similarity to a normal human ileum [42].

Conventional monolayer and organic culture systems maintained in a non-physiological environment do not provide a realistic overview of the in vivo function, intestinal microstructure, and the formation of gradients across geometric structures (e.g., oxygen, pH, growth factors, bacteria) [64,65], such that functional readouts (e.g., integrated biosensor or ELISA tests) help to assess the barrier integrity, oxygen concentration, and inflammatory response [66,67]. Surprisingly, these challenges have recently been overcome by the advent of high-fidelity complex gut microarray models in which stable aerobic and anaerobic microbial communities derived from human stool samples are co-cultured with live human intestinal epithelium under a hypoxic gradient similar to that observed in vivo [44]. Firoozinezhad et al. [44] demonstrated the extended co-culture of live human intestinal epithelium with stable aerobic and anaerobic human gut microbial communities using microfluidic gut microarrays, allowing for a controlled and real-time assessment of physiologically relevant oxygen gradients. GOC provides control over many system parameters that contribute to the study of a wide range of physiological phenomena.

## 3. Gut–Organ-Axis on Chip

### 3.1. Gut–Brain Axis (GBA) on Chip

**Figure 4:**
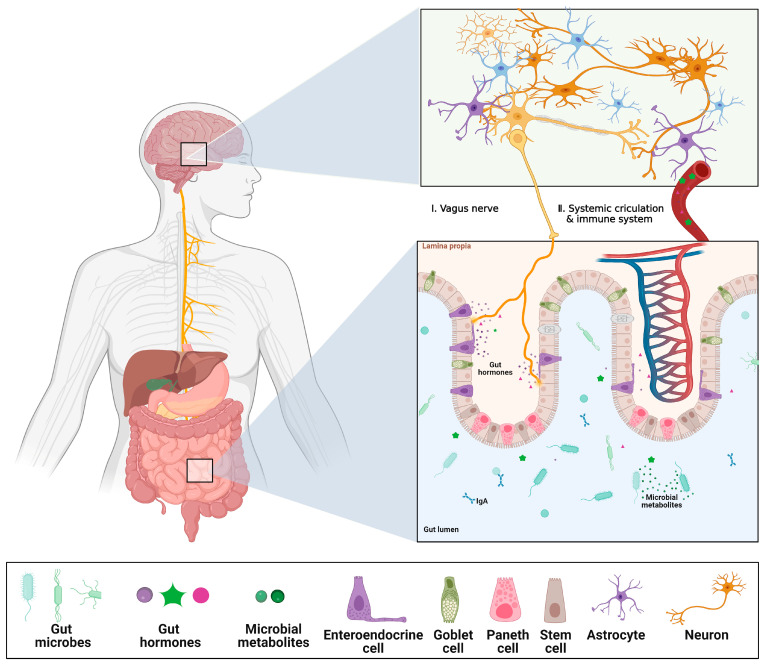
Schematics of Gut–Brain Axis (GBA). The gut luminal environment, including the gut microbiome, affects the physiology and behavior of the brain via multiple routes. (**I**) The vague nerve senses gut hormones and microbial metabolites from the gut environment and delivers the signals to the brain. (**II**) Microbial products can cross the gut epithelial barrier, which makes them eventually enter systemic circulation. Additionally, some gut hormones are secreted into the bloodstream, stimulating the immune system, or traveling to the BBB, which affects changes in the physiology and behavior of the BBB and brain. Created with BioRender.com. The reproduction of this image has been licensed from BioRender.

Research has verified that the brain and gut can “talk” directly to each other, forming the gut–brain axis (GBA), indicating that the gut environment may affect the neurocognitive function of the brain [68,69]. Perception between the gut microbiota and the CNS happens mainly through microbially-derived intermediates, such as common SCFAs, secondary biliary acids (2BAs), and tryptophan metabolites [70]. While some of these intermediates interact directly with intestinal endocrine cells, intestinal villi cells, and the mucosal immune system to propagate “bottom-up” signals, others cross the intestinal barrier into the blood circulation and may even cross the Blood–Brain barrier (BBB) [70,71,72]. The development of degenerative brain diseases such as Alzheimer’s disease or Parkinson’s disease has been linked to gut health [73,74]. Using multi-OOC to reproduce the brain–gut-immune axis across organs allows for the study of how these organ systems evolve in response to each other. The GBA has two main barriers, the gut barrier and the BBB, which are frequently exposed to fluid flow in the body. It is thought that the gut and brain communicate through multiple pathways, one of which is the delivery of soluble microbial derivatives from the microbiota to brain cells through the gut epithelium and the BBB [75,76]. The intestinal epithelium safeguards the body’s circulation from hazardous foreign compounds, whereas the BBB plays an essential role in maintaining the physical and chemical balance of the brain and in protecting it from harmful molecules and pathogens in the blood [77,78,79]. Some substrates or membrane vesicles originating from the intestinal environment, such as exosomes, may reach the BBB through the bloodstream and ultimately influence the brain (Figure 4).

**Figure 5:**
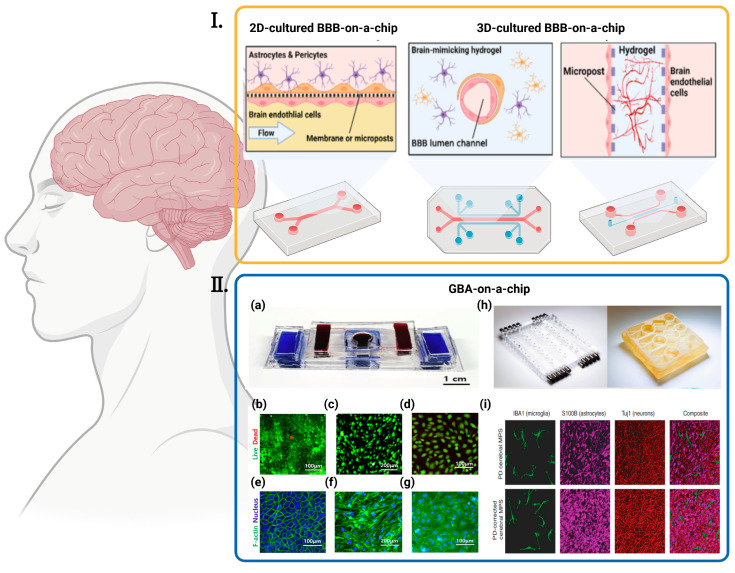
Various BBBs-on-a-chip and GBA-on-chip. (**I**) Strategies for reconstructing the in vitro BBB in an OOC platform are diverse. Cells can be cultured in a 2D environment under fluidic flow or 3D-cultured with different approaches, such as seeding in hollow hydrogel or the angio/vasculogenesis approach [27] ©Copyright 2020, MDPI. (**II**) Various GBA-on-chip. (**a**) The picture of assembled GBA chip [30] ©Copyright 2020, Elsevier; (**b**–**g**) Fluorescent images of cells seeded in the chip [30] ©Copyright 2020, Elsevier: (**b**) Live/Dead Images of Caco-2, (**c**) bEnd.3, and (**d**) hBMECs when co-cultured (green = live, red = dead). F-actin/nucleus stain images of (**e**) Caco-2 cells, (**f**) bEnd.3 cells and (**g**) hBMECs when co-cultured (blue = nucleus, green = F-actin); (**h**) Left: pneumatic plates machined in acrylic; Right: mesofluidic plate machined from monolithic polysulfone [82] ©Copyright 2021, Amer Assoc Advancement Science; (**i**) Representative, 3D rendered confocal images of the PD (top) and control PD-C (bottom) cerebral MPSs composed of hiPSC-derived microglia (green), astrocytes (purple), and neurons (red) cocultured on 0.4-μm microporous 24-well Transwells [82] ©Copyright 2021, Amer Assoc Advancement Science.

Due to its complexity, studies of GBA rely heavily on in vivo animal models [75]. They have a poor experimental reproducibility [80] and they make it difficult to perceive responses in real-time [18]. Moreover, the extrapolation of animal data to humans can be questionable, and a detailed investigation of the underlying mechanisms is often difficult, leading to an increased need for in vitro experimental models for GBA studies. Recent advances in GBA microarray technology may be one way to address these issues [30]. To our knowledge, few GBA on a chip have been reported and we currently lack a realistic platform to study inter-organ communication between the gut and the brain [81]. In their review paper, Raimondi et al. provide a blueprint for the GBA-on-a-Chip [75] (Figure 5).

Wang et al. devised a chip design in which two adjacent barrier structures can be placed in close proximity to each other while being exposed to a controlled fluid flow [81]. The chip was developed as a modular GBA chip based on the research of Lee et al. [83]. The microfluidic device consists of two parts: an intestinal barrier module (upper part) and a BBB module (lower part). After the co-culture of intestinal epithelial cells with brain endothelial cells, the barrier was observed to change by measuring trans endothelial/epithelial resistance (TEER) after LPS or butyrate treatment. The transport of exosomes through the intestinal barrier to the BBB was also investigated. The responses to microbial homologs on the chip and in the well were compared and they were enabled to generalize the reported effects of these substrates. The chip may become a new in vitro platform for studying inter-organ communication between the gut and the brain [81].

Kim et al. developed a modular microfluidic chip in which intestinal epithelial cells and brain endothelial cells were co-cultured to create the intestinal epithelial barrier and the BBB and interconnected via microfluidic channels. The modules are easy to assemble and disassemble, are co-cultured under an appropriate fluid flow, and the cell barriers are well-formed. The response to microbial by-products was consistent with previously known observations of intestinal epithelial cell and blood–brain barrier interactions, where they observed the transport of fluorescently labeled exosomes across the intestinal barrier to the BBB [30]. Recently, Trapecar et al. constructed a modular GBA chip to model gut–liver–brain interactions [82]. Under this platform, they assembled a main body circulatory system that connects each module in the axial gut–liver–brain circulatory route. Using this platform, they successfully modeled in vivo behavior in the brain module and found that microbiome-associated short-chain fatty acids increased the expression of pathology-related pathways in Parkinson’s disease patients [82]. Unfortunately, the model has several limitations. Firstly, the platform lacks direct exposure of the gut module to shear, which can affect the physiological response of the gut in vitro, and secondly, the researchers did not incorporate the blood–brain barrier into their model, which is another important hurdle in the context of GBA.

It is well known that communication between the gut microbiota and the CNS occurs primarily through microbial-derived intermediates, most commonly SCFAs, secondary bile acids (2BAs), and tryptophan metabolites, which are also commonly detected as “signals” [84]. At the same time, the CNS can influence the intestinal flora directly through the intraluminal secretion of endocrine mediators that interact with microbial receptors, and indirectly through the regulation of the intestinal environment [85]. Direct signals usually involve catecholamines, the concentration of which can be influenced by physiological and psychological stress, while indirect signals involve both branches of the autonomic nervous system (ANS) [85]. In mouse models of brain injury, an increased release of norepinephrine leads to a reduction in the number of cupped cells and the production of mucin, which leads to changes in the gut microbiota that correlate with the degree of injury. In the future development of gut–organ-axis microarrays, these signals observed in the “mini-brain and mini-gut” could be used to infer the in vivo regulation of the gut–brain axis, thus providing further possibilities for future applications of gut–brain microarrays in brain-related diseases.

### 3.2. Gut–Liver Axis (GLA) on Chip

**Figure 6:**
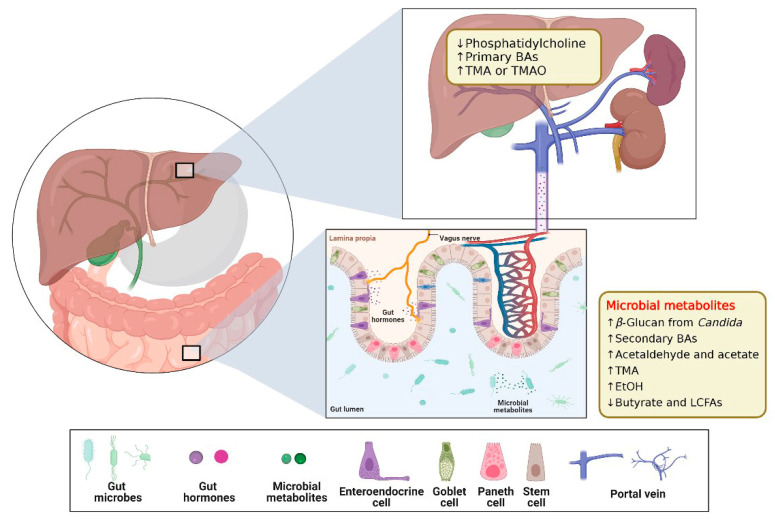
Schematics of Gut–liver Axis (GLA). The gut luminal environment, including the gut microbiome, affects the physiology and behavior of the liver via multiple routes. Created with BioRender.com. The reproduction of this image has been licensed from BioRender.

Intestinal and hepatic microphysiological systems can be associated with model intestinal barrier disruption and liver injury (Figure 6) in response to autoimmune and inflammatory drivers of the gut–liver axis (GLA) [86]. The intestine and liver communicate closely in both directions through the biliary channel, the portal vein, and the systemic circulation. The liver communicates with the gut by emitting bile acids and numerous bioactive mediators into the biliary tract and the systemic circulation [87]. In the gut, hosts and microorganisms metabolize endogenous substrates (e.g., bile acids and amino acids) as effectively as exogenous substrates (from diet and environmental exposure), the products of which are transferred to the liver through the portal vein and affect liver function [10].

**Figure 7:**
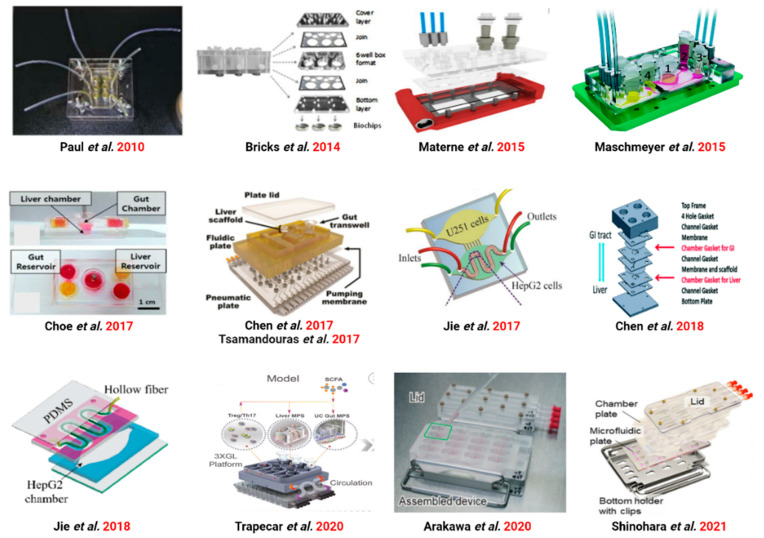
Various GLA-on-chip from 2010 to 2021. Ref. [88] ©Copyright 2010, Royal Soc Chemistry. Ref. [89] ©Copyright 2014, Pergamon-Elsevier Science Ltd. Ref. [90] ©Copyright 2015, Amer Chemical Soc. Ref. [58] ©Copyright 2015, Royal Soc Chemistry. Ref. [56] ©Copyright 2017, Springer. Ref. [33] ©Copyright 2017, Wiley. Ref. [91] ©Copyright 2017, Springer. Ref. [92] ©Copyright 2017, Royal Soc Chemistry. Ref. [93] ©Copyright 2018, Royal Soc Chemistry. Ref. [94] ©Copyright 2018, Royal Soc Chemistry. Ref. [86] ©Copyright 2020, Cell Press. Ref. [35] ©Copyright 2020, Royal Soc Chemistry. Ref. [34] ©Copyright 2021, Science.

The intestine and liver are the main barriers to the first metabolism of orally consumed foods, drugs, and other substances and their capability to regulate drug transport greatly influences the concentration available in the body and regulates the efficacy and side effects of drugs and the appearance of food nutrients. In current in vitro models, it is difficult to study the multi-organ nature of first-pass metabolism using traditional cell culture methods. GBA microarrays, however, can address these problems faced. We summarized the existing studies of GBA microarrays (Figure 7).

#### 3.2.1. GLA Chip of Physiological Mechanisms Studies

**Figure 8:**
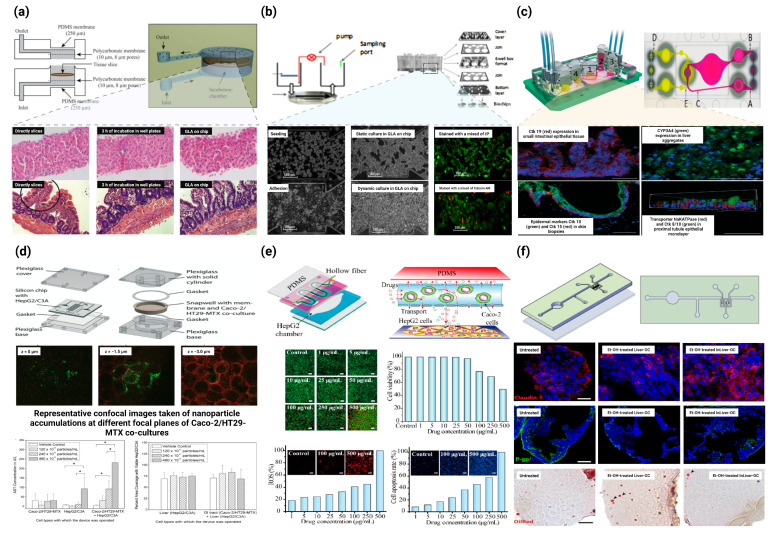
Application of GLA-on-chip in drug experiments and disease models. (**a**) A microfluidic approach for in vitro assessment of inter-organ interactions in drug metabolism using GLA-on-chip. Top: Schematic illustration and photograph of the PDMS biochip. Bottom: Morphological evaluation of liver and intestinal slices directly after slicing, after 3 h of incubation in well plates, and in the biochip. (Magnification: 100×) [88] ©Copyright 2010, Royal Soc Chemistry. (**b**) Development of a new microfluidic platform integrating co-cultures of intestinal and liver cell lines. Top: Principle, design of GLA-on-chip. Bottom: Microscopic analysis of HepG2 C3A integrity in the microchips [89] ©Copyright 2014, Elsevier. (**c**) A four-organ-chip for interconnected long-term co-culture of human intestine, liver, skin, and kidney equivalents. Top: The microfluidic four-organ-chip device at a glance. Bottom: Performance of human tissues in the 4 °C after 28 days of co-culture [58] ©Copyright 2015, Royal Soc Chemistry. (**d**) Body-on-a-chip simulation with gastrointestinal (GI) tract and liver tissues suggests that ingested nanoparticles have the potential to cause liver injury. Top: Schematic of the silicon chip and GI tract module of the body-on-a-chip system. Middle: Representative confocal images taken of nanoparticle accumulations at different focal planes of Caco-2/HT29-MTX co-cultures. Bottom: Mean concentrations of aspartate aminotransferase (AST) and the percent area of on-chip liver chambers that was covered with viable HepG2/C3A cells after 24 h of exposure to 50 nm carboxylate polystyrene nanoparticles at varying concentrations [97] ©Copyright 2014, Royal Soc Chemistry. (**e**) GLA-on-chip for multiple drugs absorption and metabolism behavior simulations. Top: Schematic illustration of the double-layer microchip. Bottom: Influence of genistein and dacarbazine combination influence on HepG2 cell viability and apoptosis in the intestine–liver model [94] ©Copyright 2018, Science Press. (**f**) GLA-on-chip reveals the intestinal protective role on hepatic damage by emulating ethanol first-pass metabolism. Top: Schematic representation of the InLiver-OC and CFD study. Bottom: Protective role of 3D-HIM on Et-OH-induced liver cytotoxicity [98] ©Copyright 2020, Frontiers. * represents significance analysis, * *p* < 0.05.

In 2010, Paul et al. combined the liver and intestine in a microfluidic device for the first time [88] (Figure 8a). Intestinal and liver sections functioned on the microarray and were shown to be applicable to organ interactions, including the regulation of bile acid synthesis. The system enabled in vitro studies possible and provides insights into organ–organ interactions [88]. Thereafter, many organs are grouped together on a single chip.

GLA microarrays are also widely used to establish applications for liver-related diseases. Hepatic steatosis is a process of abnormal lipid deposition within hepatocytes, commonly caused by excessive alcohol intake or obesity [95]. A traditional in vitro model of hepatic steatosis uses hepatocytes in culture, treated with fatty acids, and measure the intracellular lipid accumulation. This model does not outline the complex processes involved in the uptake and metabolism of digested lipids. Here, Lee et al. proposed a gut–liver-on-chip that simulated intestinal absorption and hepatic metabolism in a microfluidic chip and demonstrated that fatty acids are absorbed through the intestinal layer and subsequently deposited in hepatocytes. Researchers selected TNF-α, butyric acid, and α-lipoic acid as model molecules for the different mechanisms affecting hepatic steatosis and evaluated their effects. The results indicated that the gut–liver-on-chip can mimic the absorption and accumulation of fatty acids in the intestine and liver [83]. Trapecar et al. created a human multi-organ model of ulcerative colitis by containing circulating Treg and Th17 immune cells connected by fluid circulation [86]. This model was combined with multi-omics and the results showed that SCFAs ameliorated or worsen the disease and were determined by CD4^+^ T cell effector functions. This study uniquely demonstrates how human multi-organ systems can be used to better understand the immune and metabolic regulation of human pathophysiology [86]. Duan et al. developed a membrane-free liver–gut microarray platform to study PM2.5-induced metabolic disorders. The cellular metabolic mechanisms triggered by PM2.5 exposure were summarized by them: disruption of hepatic cholesterol metabolism and the disruption of primary and secondary bile acid biosynthesis [90].

Shinohara et al. co-cultured human-induced pluripotent stem cell-derived intestinal cells with fresh human hepatocytes isolated from PXB mice in a pneumatic multi-organ micro physiological system (MPS) with a pipette. This study demonstrates for the first time the co-culture of hiPS intestinal cells and fresh human hepatocytes on the MPS to detect purely inter-organ interactions [34].

#### 3.2.2. GLA Chip for Drug Metabolism Studies

In 2014, Bricks et al. generated a reusable liver–intestinal co-culture system using a 2D membrane design that recapitulated the uptake and metabolism of acetaminophen (Figure 8b). Thereafter, many organs are grouped together on a single chip. Throughout the culture process, the organ chips need to maintain a stable fluid connection, avoid bacterial contamination, and monitor cell viability. As the number of organs on the chip increases, the complexity of the system increases, inevitably leading to unpredictable results. A simplification of existing systems is essential to enable a wider range of applications [89]. Hepatocytes cultured in the microfluidic system were markedly better at metabolizing finasteride compared to the static 2D co-culture of Trans wells. In a follow-up study by Maschmeyer et al. in 2015, their model design was extended to accommodate four organs, including the liver, skin, kidney, and intestine [58] (Figure 8c). Similar models of the liver–intestine axis have been developed to allow for cell recovery after co-culture and to assess drug transport through the intestinal epithelium and its subsequent metabolism in hepatocytes. Skandal et al. developed an on-chip transfer platform that uses fluorescence to track colon cancer cells from hydrogel intestinal structures and uses a circulating fluid device to observe the invasion of downstream liver structures [96]. Metastatic foci grow and eventually disseminate from intestinal structures, enter the circulation, and subsequently reach the liver structures. Although the hepatic-intestinal system is primarily used to study metabolism and inter-organ communication, these models are also expected to provide insight into the behavior of invasive and metastatic cancers (Figure 8d).

Choe et al. created a visceral-liver-on-a-chip platform that can simulate the kinetics of first-time metabolism [56]. Caco-2 cells were employed as an intestinal epithelium model and HepG2 cells were utilized as a liver model. In order to resume the process of absorption and metabolism, fluid entered the liver lumen from the intestinal lumen. Notably, the co-culture of cells resulted in changes in the physiological functions of both cell types. Under shear stress, the permeability of Caco-2 cells was observably reduced and both cell lines exhibited increased cytochrome P450 activity and markedly elevated metabolic activity. The remarkable alterations in the behavior of two cell types affirm the need to develop co-culture systems to augment the physiological relevance of in vitro models. Other models of the intestine–liver relationship have been developed for the administration of specific treatments, including the ability of the intestine to protect the liver from nanoparticle invasion [97]. Chen et al. created a liver–gut system demonstrating that the gut can genetically control the production of bile by the liver. Furthermore, under inflammatory conditions, these two organs communicate to amplify the inflammatory response [33]. Tsamandouras et al. proposed an alternative MoC in vitro system to study the different pharmacokinetic processes associated with oral administration in humans, including intestinal permeability and hepatic metabolism [91]. Their study showed that inter-organ communication can up-regulate liver metabolism.

Arakawa et al. first developed a pharmacokinetic model of triazolam (TRZ) and its metabolites in a two-organ entero–hepatic MPS consisting of intestinal Caco-2 cells and hepatic HepaRG cells and attempted to extrapolate the kinetic information obtained from the MPS to the human blood concentration profile. In two-organ MPS and HepaRG monoculture systems, TRZ was metabolized to *α*- and 4-hydroxytriazolam and their respective glucuronides. All these metabolites were almost completely reduced in the presence of the CYP3A inhibitor itraconazole, confirming sequential phase I and phase II metabolism. Pharmacokinetic model-dependent and non-dependent analyses of the metabolic activity of TRZ provided consistent results: the clearance of glucuronidated metabolites was higher in the two-organ MPS than in the monoculture system. The distribution of blood concentrations of TRZ and its two hydroxyl metabolites in humans was quantitatively modeled based on pharmacokinetic models by introducing several scaling factors representing the quantitative gap between MPS and humans [35]. De Gregorio used a bottom-up tissue engineering strategy to construct physiologically functional 3D human gut models (3D-him) and 3D liver microscopic tissues (HepG2-µ- TPs) in vitro, and designed a microfluidic gut–liver-on-a-chip (InLiver-OC) to mimic the first-pass mechanism that occurs in vivo (Figure 8f). This study highlighted ethanol-induced 3D-him hyperpermeability and interstitial injury, the prevention of liver injury by the gut, and the synergistic contribution of both 3D tissue models to the metabolic enzyme release following a high-dose ethanol administration [98].

#### 3.2.3. GLA Chip for Drug Toxicity Studies

Jie et al. developed a gut–liver–glioblastoma biomimetic system to evaluate drug combination therapy for glioblastoma. A hollow fiber (HF) was inserted into the upper layer of a chip and used to culture Caco-2 cells as an artificial intestine to simulate drug delivery. HepG2 cells cultured on the substrate of the chip were utilized as artificial livers to metabolize the drug [92]. A two-drug combination for glioblastoma U251 cells was appraised based on an entero–hepatic metabolic model. Drugs such as irinotecan (CPT-11), temozolomide (TMZ), and cyclophosphamide (CP) were dynamically stimulated by continuous injection into the intestinal unit. Following intestinal absorption and hepatic metabolism, the prodrugs were converted into active metabolites that induce apoptosis in glioblastoma cells. The metabolic mechanisms of CPT-11 and TMZ were further investigated by combining them with mass spectrometry analysis (Figure 8e).

To demonstrate the potential of the GIT–liver model to predict the human response in preclinical studies, Chen et al. designed an integrated system in which primary human intestinal cells (hIECs) were cultured together with 3D liver tissue [93]. The hIECs were immortalised by transducing the cells with hTERT and they were subsequently connected to the liver tissue via gravity-driven media flow between their respective chambers. Both hIECs and HepG2 C3A hepatocytes exhibited good survivability after 14 days of co-culture. By measuring the uptake of caffeine, mannitol, and propranolol, the investigation continued to compare the permeability of the gut model with that of the Transwell model using Caco-2 cells. This gut–liver microchip system produced consistent metabolic rates after 2 weeks of testing, as urea and albumin production were consistent throughout the system. Compared to the single OoC system, the co-culture gastrointestinal–liver system showed significantly higher metabolic enzyme CYP activity [93]. To study the absorption and metabolism of compound drugs, Jie et al. developed an HF-based bilayer microfluidic chip to reconstruct the hepatic and intestinal functions. The results indicated that the combined concentration below 100 μg/mL had no apparent inhibitory effect on HepG2 cell viability, and hence, HepG2 cells preserved their metabolism of the drug. At drug concentrations higher than 250 μg/mL, HepG2 cells underwent apoptosis. The metabolites were detected by mass spectrometry and proved to be correct in the microarray model. This dynamic co-culture microarray successfully provided a platform for a long-term observation of the uptake, transport, and metabolism of the combined drug, providing an effective in vitro simulation model for further clinical studies [94].

### 3.3. Gut–Kidney Axis (GKA) on Chip

**Figure 9:**
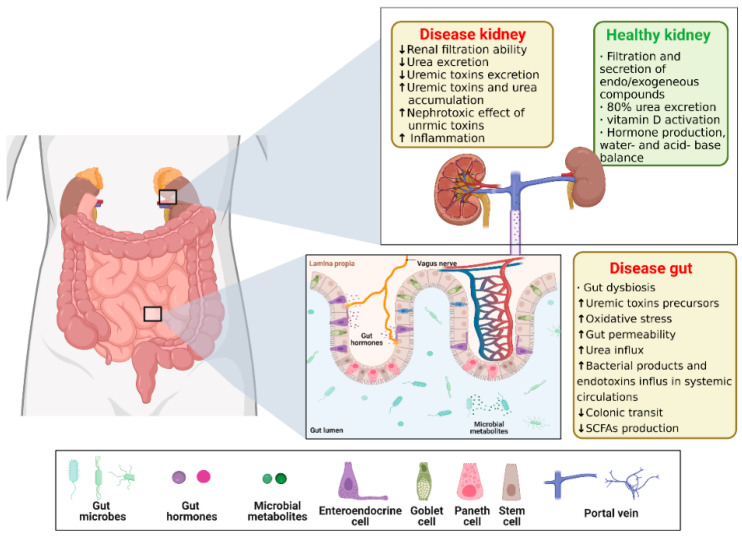
Schematics of Gut–kidney Axis (GKA). The gut luminal environment, including the gut microbiome, affects the physiology and behavior of the kidney via multiple routes. Created with BioRender.com. The ↑ and ↓ arrows in the graph represent an increase or decrease in content, performance, and/or activity, respectively. The reproduction of this image has been licensed from BioRender.

Chronic kidney disease (CKD) affects approximately 10% of the world’s population and has an annual economic impact of approximately 48 billion in the United States alone [99]. Toxin accumulation from renal disease affects the intestine [12], and imbalances in the intestinal flora and function, in turn, affecting the metabolic and toxic excretion processes of the kidney, and interacting to form a vicious circle [100]. Indeed, SCFAs, especially butyric acid, are both nephroprotective and gut-protective, and high levels of butyric acid are associated with an improved intestinal barrier integrity and intestinal immunity due to their anti-inflammatory properties [101,102]. Therefore, there is an urgent need to develop a micro-model of the gut–kidney axis chip to uncover the kidney–gut interaction process. Stark examples such as entero–kidney microarrays with multiple interfaces and compartmentalized microchambers have been used to effectively assess drug absorption-related nephrotoxicity [103], but the development of single kidney-on-a-chip systems is also challenging owing to the lack of functional cells to encapsulate the complexity of multicellular structure and function within the kidney unit in vitro. As a result, the development of the kidney-on-a-chip system has lagged to somewhat relative to the gut-on-a-chip device. To date, models of glomerular, proximal tubular, and distal tubular physiology have been developed, but the integration of all components into a complete kidney-on-a-chip unit remains to be achieved [104]. In chronic kidney disease, a decrease in SCFA production is coupled with a concomitant increase in uremic toxin production and systemic accumulation [101,105] (Figure 9).

**Figure 10:**
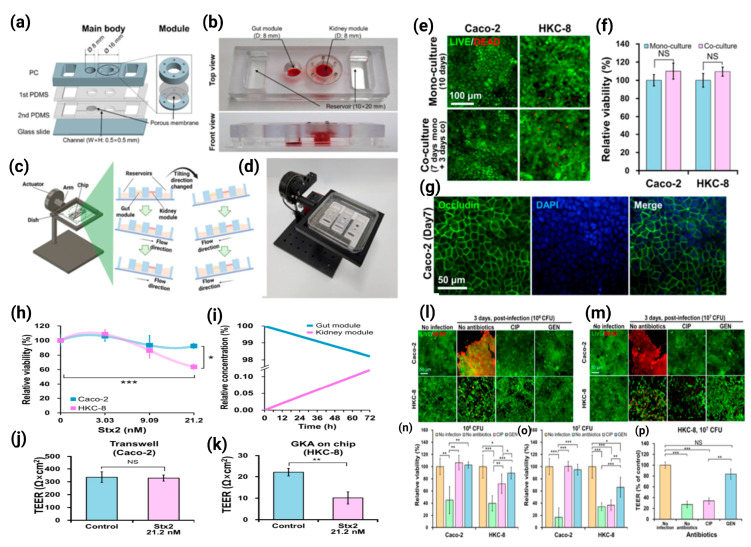
GKA on chip for studying effects of antibiotics on risk of hemolytic uremic syndrome by Shiga toxin-producing *Escherichia coli.* (**a**) Assembly of a main body in four layers with modules. (**b**) View of completed GKA on chip showing gut and kidney modules and two reservoirs. (**c**) Gravity-induced perfusion by periodically tilting the chip 10 degrees (0.1 degree/s) every 10 min. (**d**) View of the tilting machine inducing gravity-driven perfusion of cell culture medium in GKA on chip. (**e**) LIVE/DEAD stained images and (**f**) EZ-CytoX assay of Caco-2 and HKC-8 cells either mono-cultured or co-cultured for 3 days. (**g**) Immunostaining of occludin in Caco-2 cells at day 7. (**h**) Viabilities of Caco-2 and HKC-8 cells to Stx2 in GKA on chip. Only the gut module was treated with the toxin at different concentrations (0–21.2 nM) for 72 h. (**i**) Simulation of Stx2 transport from the gut module to the kidney module in the Experimental Section. (**j**) TEER Value of Caco-2 cells to Stx2 at 21.2 nM in Transwell for 72 h. (**k**) TEER values of HKC-8 cells after treatment with 21.2 nM of Stx2 in the gut module for 72 h. Sample number n= 3, Student’s *t*-test. NS; not significant, * *p* < 0.05, ** *p* < 0.01, *** *p* < 0.001. (**l**,**m**) LIVE/DEAD stained images from 10^6^ and 10^7^ CFU per module and (**n**,**o**) cell viability of Caco-2 and HKC-8 cells being treated with O157 lysed by either CIP or GEN in the chip for 72 h. The relative viability was calculated as the number of viable cells divided by the number of viable cells in the control (no infection and no antibiotics) from LIVE/DEAD stained images. (**p**) TEER values of HKC-8 cells in module of the chip when the gut module was infected with O157 at 10^7^ CFU and treated with either CIP or GEN for 72 h. n = 3, Student’s t-test, NS; not significant, * *p* < 0.05, ** *p* < 0.01, *** *p* < 0.001. [32] ©Copyright 2021, MDPI.

Lee et al. developed the intestine–kidney axis (GKA) microarrays to co-culture intestinal (Caco-2) and renal (HKC-8) cells and observed a STEC O157:H7 (O157) infection and Shiga toxin (Stx) toxicity in the intestinal and renal cells, respectively, on the microarrays. In the absence of any antibiotic treatment, O157 killed intestinal and renal cells in GKA on microarrays. CIP treatment reduced the intestinal cell O157 infection, but increased the stx2-induced renal cell injury, whereas gentamicin treatment reduced both the intestinal cell O157 infection and stx2-induced renal cell injury. This is the first report to outline the clinical correlation that CIP treatment caused more damage than gentamicin treatment. These results suggest that the entero–kidney axis (GKA) microarray was useful for simultaneously observing the O157 infection and stx2 toxicity in intestinal and renal cells and was suitable for studying the impact of antibiotics on the risk of hemolytic uremic syndrome (HUS) [32] (Figure 10).

### 3.4. Gut–Lung Axis (GLAx) on Chip

**Figure 11:**
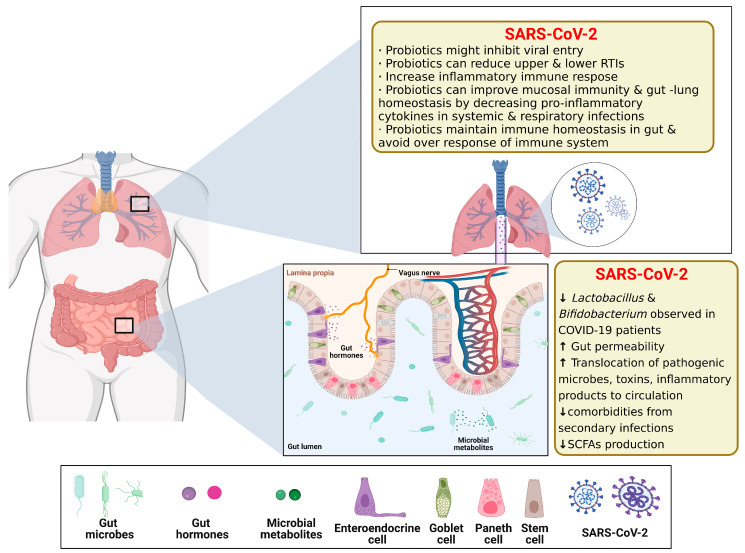
Schematics of Gut–lung Axis (GLAx). The gut luminal environment, including the gut microbiome, affects the physiology and behavior of the lung via multiple routes. Created with BioRender.com. The ↑ and ↓ arrows in the graph represent an increase or decrease in content, performance, relative abundance, and/or activity, respectively. The reproduction of this image has been licensed from BioRender.

Recent studies have shown that various chronic lung diseases, including asthma, chronic obstructive pulmonary disease (COPD), and cystic fibrosis, are closely associated with dysbiosis of the airway microflora [106,107]. This is usually the result of a loss of bacterial diversity due to the growth of certain pathogenic bacteria [14]. The airway microbiota of patients with chronic lung disease exhibits a disease-specific phenotype. Compared to healthy individuals, patients with asthma are overrepresented in the phylum *Aspergillus* (especially *Haemophilus*, *Moraxella,* and *Neisseria*) and the *Firmicutes* (*Lactobacillus*), while the phylum *Proteus* (especially *Prevotella*) is significantly under-represented [14,106]. Pulmonary complications such as parenchymal lesions can also occur in patients with IBD or Crohn’s disease, and intestinal pulmonary schistosome infections can migrate to pulmonary infections [108], especially in the treatment of Coronavirus disease 2019 (COVID-19), where the balance of the intestinal microecology is important. [109]. The microbiota plays a fundamental role in the development and function of the immune system, both locally and systemically. New experimental and epidemiological evidence highlights a critical interaction between the gut microbiota and the lung, referred to as the “gut-lung axis” (Figure 11). Changes in the gut microbiota composition through diet, disease, or pharmacological interventions (e.g., antibiotics) have been associated with alterations in respiratory immune responses and endostatins. The relevance of the gut–lung axis gained even greater prominence after some gut microbial-derived components and metabolites, such as SCFAs, were identified as key mediators in determining the immune system tone [14,110].

**Figure 12:**
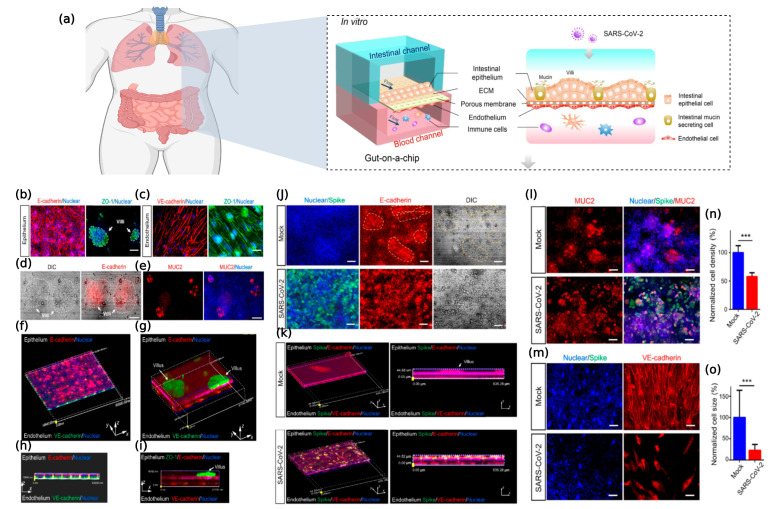
SARS-CoV-2-induced intestinal responses with a GLA-on-chip. (**a**) The configuration of the multilayered intestine on the chip device infected with SARS-CoV-2. (**b**) Confocal micrographs of the intestinal epithelial barrier on the chip visualized by the expression of an adhesion junction (E-cadherin) and tight junction markers (ZO-1). The intestinal villus-like structures with high levels of ZO-1 expression are indicated by white dashed lines. (**c**) Confocal micrographs of the vascular endothelium identified by the expression of an adhesion junction protein (VEcadherin) and ZO-1. (**d**) DIC image of an intestinal villus-like structure with clumps of cells (indicated by white dashed lines). (**e**) Immunostaining of a mucin marker (MUC2) in intestinal epithelial cells. Scale bars: 50 lm. (**f**,**h**) The 3D reconstructed confocal image and side view of the human intestinal epithelium (E-cadherin) and endothelium (VE-cadherin). (**g**,**i**) The 3D reconstructed confocal image and side view of the intestinal epithelium, endothelium, and intestinal villus-like structures (indicated by white arrows). (**j**) Confocal micrographs of SARS-CoV-2 infection (Spike protein) on the intestinal epithelium (E-cadherin) and intestinal villus-like structures (indicated by yellow dashed lines) at day 3 post-infection. Scale bars: 50 lm. (**k**) The 3D reconstructed confocal image and side view of a mock-infected gut-on-chip. The 3D reconstructed confocal image and side view of the virus-infected intestinal model. SARS-CoV-2 infection was identified in the epithelial layer by the expression of the viral Spike protein. (**l**) Confocal micrographs of SARS-CoV-2 infection (Spike protein) and MUC2 expression in the intestinal epithelium at day 3 post-infection. Scale bars: 50 lm. (**m**) Confocal micrographs of viral infection (Spike protein) in the vascular endothelium (VE-cadherin). Scale bars: 50 lm. (**n**,**o**) Quantification of endothelial cell density and size for mock- and SARS-CoV-2-infected chips. Four chips were counted for cell density quantification in each group, and 100 cells were counted for cell size quantification in each group. Data are presented as the mean ± SD and were analyzed by Student’s t-test (***, *p* < 0.001) [31] ©Copyright 2021, Elsevier.

COVID-19, caused by severe acute respiratory syndrome coronavirus type 2 (SARSCoV-2), has become a global pandemic. Clinical evidence suggests that the intestine is another organ at high risk for SARS-CoV-2 infection besides the lungs [111]. However, models that accurately reflect the human pulmonary–intestinal axis response to the virus are still lacking. Guo et al. created an intestinal infection model on a microchip that can reproduce the SARSCoV-2-induced human-associated intestinal pathophysiology at the organ level. The intestinal epithelium was susceptible to viral infection, with marked morphological changes following intestinal villi injury, dispersed distribution of mucus-secreting cells, and a reduced expression of tight junctions (E-cadherin), suggesting the virus-induced disruption of intestinal barrier integrity. Moreover, the morphology of the vascular endothelial cells was abnormal and the attachment junctions were broken. Transcriptional analysis indicated abnormal RNA and protein metabolism in the epithelial and endothelial cells, as well as activation of the immune response (e.g., cytokine gene upregulation) following a viral infection, which may lead to intestinal barrier damage associated with gastrointestinal symptoms. This human organ system could partially reflect intestinal barrier damage and the human response to a viral infection, which is not possible in existing in vitro culture models. It provides a unique and rapid platform to accelerate COVID-19 research and the development of new therapies [31] (Figure 12).

### 3.5. Others

**Figure 13:**
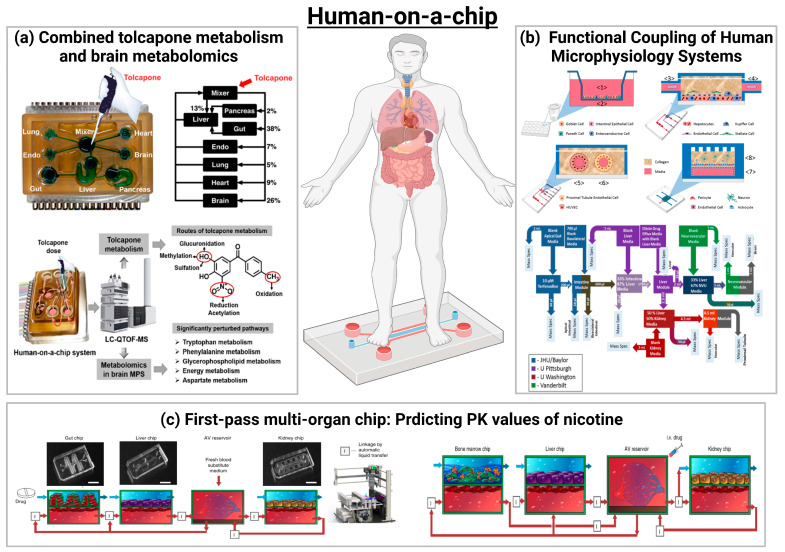
Schematics of human-on-chip. (**a**) Analysis of an Integrated Human Multi-organ Microphysiological System for Combined Tolcapone Metabolism and Brain Metabolomics [114] ©Copyright 2019, Amer Chemical Soc. (**b**) Functional Coupling of Human Microphysiology Systems: Intestine, Liver, Kidney Proximal Tubule, Blood–Brain Barrier and Skeletal Muscle [55] ©Copyright 2017, Nature Research. (**c**) Quantitative prediction of human pharmacokinetic responses to drugs via fluidically coupled vascularized organ chips [113] ©Copyright 2020, Nature Research.

In addition to the gut–organ-axis chip, the modular nature of the device on a chip makes it possible to connect multiple chips to create a multi-organ chip system, also known as a “body on a chip” [52,53,54,55]. These MoC systems, which use the gut as the primary organ-linked vehicle, can be used to study inter-organ communication, systemic pathology, pharmacology, and pathogen invasion and distribution. This is particularly useful for studying secondary toxicity caused by the metabolism of drugs in organs such as the gut and liver [56,57]. Although the OoC system can be used independently, each organ model plays a unique role in the physiologically relevant body-on-a-chip model [55,112,113,114] (Figure 13).

In 2017, Vernetti et al. [55] assessed the functional coupling of five human MPS models (intestine, liver, proximal renal tubule, BBB, and skeletal muscle), defined as organ interactions determined by an in vivo class sequential, organ-to-organ mediator transfer. In addition to skeletal muscle and neurovascular models, MPS models were evaluated representing the major organs of absorption, metabolism, and clearance (jejunum, liver, and kidney). Three compounds were appraised for their organ-specific handling: the pharmacokinetics (PK) and toxicity of terfenadine, trimethylamine (TMA) as a potentially toxic microbial metabolite, and vitamin D3. The results demonstrated that the organ-specific processing of these compounds was consistent with the clinical data and TMAO was found to cross the BBB. These studies provide evidence of the multi-organ toxicity and absorption, distribution, metabolism, and excretion (ADME) potential of human MPS, providing guidance on the physical coupling of MPS and a method for coupling MPS to different media and perfusion requirements [55].

In 2019, Wang et al. [114] applied mass spectrometry to a complex multi-organ human microarray containing seven interacting microphysiological systems (MPSs) to comprehensively study the metabolism of tolcapone by metabolite and metabolomics analysis. Ultimately, they identified 12 metabolites of tolcapone, three of which were newly reported, and demonstrated that oxidation, reduction, and conjugation reactions are the most important pathways of tolcapone metabolism. Moreover, non-targeted metabolomics identified significant changes in 18 key biomarkers in the human brain MPS following tolcapone administration, which were mainly associated with perturbations in the tryptophan and phenylalanine metabolism (BH4 cycle), glycerophospholipid metabolism, energy metabolism, and aspartate metabolism. This study is the first example of successfully combining drug metabolisms, metabolomics, and cellular engineering to capture complex human physiology and multi-organ interactions [114].

In 2020, Herland et al. developed the gut–liver–renal and bone marrow–liver–renal multi-organ systems to predict the PK parameters of nicotine (an oral drug to aid smoking cessation) and cisplatin (an intravenous anticancer drug), such as a maximum nicotine concentration in arteriovenous reservoirs and time to reach maximum levels, consistent with the clinical data [113]. The pharmacological response of cisplatin was further confirmed by the bone marrow–liver–renal multi-organ system: at 24 h of 160 μM administration, cisplatin did not exhibit hepatotoxicity in the liver chip, but showed bone marrow toxicity and nephrotoxicity in the bone marrow and kidney chips, respectively, reproducing the in vivo pharmacodynamics (PD) of cisplatin. This interrogator platform has been utilized to investigate the PK and PD parameters of oral and injected drugs—for example, via a connected entero–hepatic–renal microarray, in which PK parameters of orally administered nicotine (used to treat neurodegenerative diseases and IBD) were modeled, and to predict the PK parameters of intravenously administered chemotherapeutic drugs using a bone marrow–hepatic–renal microarray [113].

## 4. Future Advances in Gut–Organ-Axis-on-a-Chip: Challenges and Opportunities

The intestine is the second most powerful immune organ in the body, and there is still too much unpredictability regarding the impact of changes in the composition and the abundance of microbial populations on human health. The development of organoids has addressed the bottlenecks in traditional models (Table 1), but after more than 10 years of development, there are still obvious shortcomings and room for future development.

(1) Although some studies have been able to construct gut microarrays suitable for the survival of anaerobic bacteria, only specific types or classes of anaerobic bacteria have been tested, and there are still technical bottlenecks for testing the function of complex gut flora on the microarrays, which need to be addressed by further research.
(2) The human body is a complex whole, and although existing studies have proposed a variety of interoperable models such as the brain–intestinal axis, the liver–intestinal axis, and the kidney–intestinal axis, how to consider the crosstalk between multiple models is a hot issue that should be of concern in subsequent studies of disease occurrence and drug action. Organ-on-a-chip systems are still marginal in the pharmaceutical industry, and pharmacokinetic simulation studies, toxicology studies, pharmacodynamic studies, and the reduction of each organ corresponding to the physiological environment and physiological effects in vivo are also important in drug development.
(3) Although many researchers have sought to reduce the cost of using organ chips by finding more suitable materials and more convenient models, the specialized and cumbersome nature of chip design and manufacture also limits their large-scale use, so how can we improve their applicability by reducing the constraints such as the difficulty and cost of use from the perspective of raw material selection, template design, and chip manufacture? In addition to the finished chip and its suitability for use, the stability and repeatability of the chip quality is also a factor to be examined.
(4) In the future development of gut–organ-axis chips, the signal molecules observed in the “mini-organ and mini-gut” can be used to infer the in vivo regulation of the gut–organ-axis, thus suggesting further possibilities for the future application of gut–brain microarrays in gut–organ-axis-related diseases, the exploration of disease mechanisms and the development of new drugs.
(5) In terms of detection, the technological capabilities of the future intestinal axis microarray platform will allow real-time, in situ, and dynamic maintenance and monitoring of a large number of biological parameters such as shear stress, pH, oxygen, cytokines, as well as the use of methods such as electrochemical or optical- and fluorescence-based methods in conjunction with such organ systems on a chip. It is also combined with ELISA, PCR, and single-cell mRNA sequencing to correlate biomarker, molecular characterization, cell physiology, and histopathology of pathology.

## Figures and Tables

**Table 1:** Crosstalk on the “gut axis” associated organ reproduced in the gut-on-chip device.

Gut Axis on Chips	Cell/Organ Type	Targeted Application and Major Results	Ref.
GBA on chip	-Gut epithelial cells -Brain endothelial cells	-Co-culture creates a positive cell barrier -Reflects interactions between microbial by-products, intestinal epithelium and BBB -Transport of fluorescently labeled exosomes across the intestinal barrier to the blood–brain barrier can be observed	[30]
	-Human monocyte-derived dendritic cells and macrophages -Hepatocytes and Kupffer cells -Neurons, astrocytes, and microglia -T_reg_/T_H_ 17 cells	-A model of excellence for investigating neurodegenerative diseases -Systemic interactions enhance the in vivo-like behavior characteristics of brain micro-physiological systems -Microbial-associated SCFAs increase the expression of pathology-related pathways in Parkinson’s disease	[82]
GLA on chip	-Hepatocytes and Kupffer cells -Enterocyte, goblet cells, and dendritic cells	-Reveals the regulation of bile acid metabolism -Provides evidence for physiologically relevant intestine–hepatic crosstalk -Significant non-linear regulation of cytokine responses observed in inflammatory intestine–hepatic interactions	[33]
	-Human-induced pluripotent stem cell-derived intestinal cells and fresh human hepatocytes	-Exploring unknown physiological mechanisms of in vitro organ–organ interactions	[34]
	-Caco-2 cells -Hepatic HepaRG cells	-Exploration of the pharmacokinetic mechanism model of triazolam (TRZ) and its metabolites in GLA on chip -TRZ is metabolized to α- and 4-hydroxytriazolam and their respective glucuronides	[35]
	-Caco-2 cells -HepG2 cells	- Assessment of the metabolism of the flavonoid apigenin -The co-culture of intestinal and liver cells on the microarray led to a metabolic profile that was stronger than the monoculture effect	[56]
	-Caco-2 cells -Hepatic cells -T_reg_/T_H_ 17 cells	-Used as an in vitro model of ulcerative colitis (UC) -SCFAs can ameliorate or worsen the severity of UC, depending on the involvement of effector CD4^+^ T cells -SCFA increased ketone body, glycolysis, and lipogenesis production, while it significantly reduced innate immune activation in the UC gut	[86]
	-Caco-2 TC7 cells -HepG2 C3A	- Dynamic intestine liver coculture model -First pass metabolism of phenacetin investigation -Higher metabolic performance of the bioreactor when compared the Petri coculture	[89]
	-Human intestinal myofibroblasts -Caco-2 cells	-Simulates the first-pass mechanism that occurs in vivo -Emphasis on ethanol-induced 3D-him hyperpermeability and interstitial injury, and the preventive role of the intestine on liver injury -Simulation of metabolic enzyme release following high-dose ethanol administration	[98]
	-Caco-2 cells -HepG2 cells	-Evaluation of drug combinations for the treatment of glioblastoma -Continuous infusion of irinotecan (CPT-11), temozolomide (TMZ), cyclophosphamide (CP), and other drugs dynamically stimulate cells as an interventional drug model -After intestinal absorption and hepatic metabolism, the prodrugs are converted into active metabolites and induce apoptosis in glioblastoma cells -Enables long-term cell co-culture, drug delivery, metabolism and real-time analysis of drug effects	[92]
	-Caco-2 cells -HepG2 cells	-Investigating the effects of dynein and dacarbazine on cell viability, hepatotoxicity, and cell cycle arrest during combination drug therapy -Served as a platform model for long-term observation of the uptake, transport, and metabolism of the combined drugs	[94]
CKA on chip	-Caco-2 cells -HKC-8 cells	-As a model for investigating STEC O157:H7 (O157) infection and Shiga toxin 2 toxicity in intestinal and renal cells	[32]
GLAx on chip	-Caco-2 cells -HUVECs cells	-Creation of an intestinal infection model that allows reproduction of SARSCoV-2-induced human-associated intestinal pathophysiology at the organ level	[31]
multi-organ on chip	Intestine, liver, kidney proximal tubule, blood–brain barrier, and skeletal muscle	-Evaluated the pharmacokinetics (PK) and toxicity of terfenadine, trimethoprim, and vitamin D3 -Demonstrated the potential of multi-organ co-chips for multi-organ toxicity and absorption, distribution, metabolism, and excretion (ADME)	[55]
	Intestine, liver, kidney, and coupled bone marrow	-Exploration of the first physiological pharmacokinetic model of absorption, metabolism, and excretion of drugs through humans -Predicted pharmacodynamic parameters of oral nicotine and intravenous cisplatin	[113]
	Brain, pancreas, liver, lungs, heart, intestines, endometrium	-Evaluation of mephedrone metabolite profiles and metabolomics	[114]

## Data Availability

Not applicable.
